# Roles of Nmnat1 in the survival of retinal progenitors through the regulation of pro-apoptotic gene expression via histone acetylation

**DOI:** 10.1038/s41419-018-0907-0

**Published:** 2018-08-30

**Authors:** Hiroshi Kuribayashi, Yukihiro Baba, Toshiro Iwagawa, Eisuke Arai, Akira Murakami, Sumiko Watanabe

**Affiliations:** 1 Division of Molecular and Developmental Biology, Institute of Medical Science, University of Tokyo, Tokyo, Japan; 2 Graduate School of Medicine, Juntendo University, Tokyo, Japan

## Abstract

Leber congenital amaurosis (LCA) is a severe, genetically heterogeneous dystrophy of the retina and mutations in the nicotinamide mononucleotide adenylyltransferase 1 (*NMNAT1*) gene is one of causal factors of LCA. NMNAT1 is a nuclear enzyme essential for nicotinamide adenine dinucleotide (NAD) biosynthesis pathways, but the mechanisms underlying the LCA pathology and whether NMNAT1 has a role in normal retinal development remain unclear. Thus, we examined the roles of Nmnat1 in retinal development via short hairpin (sh)-RNA-mediated downregulation. Retinal explants expressing sh-Nmnat1 showed large numbers of apoptotic retinal progenitor cells in the inner half of the neuroblastic layer. Decreased intracellular NAD content was observed and the addition of NAD to the culture medium attenuated sh-Nmnat1-induced apoptosis. Of the nuclear Sirtuin (Sirt) family, the expression of sh-Sirt1 and sh-Sirt6 resulted in a phenotype similar to that of sh-Nmnat1. Sirt proteins are histone deacetylases and the expression of sh-Nmnat1 increased the levels of acetylated histones H3 and H4 in the retina. Expression of sh-Nmnat1 resulted in significantly increased expression of *Noxa* and *Fas*, two pro-apoptotic genes. Acetylation of the genomic 5′-untranslated regions of *Noxa* and *Fas* loci was upregulated by sh-Nmnat1 expression. The co-expression of sh-Fas with sh-Nmnat1 reduced the number of apoptotic cells induced by sh-Nmnat1 expression alone. Taken together, our data suggested that the increased expression of *Noxa* and *Fas* explains, at least in part, the phenotype associated with sh-Nmnat1 in the retina. Taken together, these findings demonstrate the importance of the NAD biosynthesis pathway in normal development of the retina.

## Introduction

Leber congenital amaurosis (LCA) is a genetically heterogeneous inherited photoreceptor dystrophy characterized by severe early-onset photoreceptor degeneration. It was first described by Theodor Leber in 1869^1,2^ and, to date, 18 mutated genes have been identified as causal genes of LCA; most LCA cases are inherited in an autosomal recessive manner^3^; [RetNet; https://sph.uth.edu/retnet/)]. From birth, LCA patients exhibit severely impaired visual function caused by the rapid degeneration of both rods and cones.

Recently, several research groups independently identified mutations of the nicotinamide (NAM) mononucleotide adenylyltransferase 1 (*NMNAT1*) gene in patients with LCA type 9 (https://www.ncbi.nlm.nih.gov/books/NBK1298/)^4–7^. NAM adenine dinucleotide (NAD) is synthesized by two different de novo pathways; one is a major pathway referred to as a salvage pathway and the other is the Preiss–Handler pathway. The NMNAT family includes enzymes essential for both of these pathways and consists of three members (NMNAT1–3) with distinct subcellular distribution patterns in mammals^8^. NMNAT1 localizes to the nucleus, whereas the other two isozymes are found in the Golgi and the cytoplasm (NMNAT2) and the mitochondria (NMNAT3)^9^. All three members catalyze the same adenosine triphosphate-dependent adenylation of NAM mononucleotide (NMN) or NAM acid mononucleotide (NaMN).

Subsequent to initial reports, several studies including our research group have identified *NMNAT1* mutations as a pathogenic cause of LCA^10–12^. Although the neuroprotective activities of NAD have been suggested to have a role in this process, especially in the case of Wallerian degeneration^13^, the mechanism by which Nmnat1 influences retinal degeneration has yet to be elucidated.

NMNAT1 is a ubiquitous enzyme and studies investigating whether NMNAT2 and NMNAT3 have redundant roles are inconclusive. For example, *Nmnat1* knockout mice die at the embryonic stage^14^, which suggests that Nmnat2 and Nmnat3 cannot compensate for the critical role of Nmnat1 during embryogenesis. Recently, mice with an *Nmnat1* missense mutation were identified in a mouse pool of chemical mutagenesis and retinal degradation, without any other apparent phenotype, was observed in these mice after birth^15^. These findings suggest that mutation of *Nmnat1* specifically affects retinal maintenance in a mouse model, as in humans.

However, even the role of Nmnat1 and/or NAD in development of retina had not been clarified. Thus, the present study aimed to analyze the role that Nmnat1 has in mouse retinal development using short hairpin (sh)-RNA-mediated downregulation of Nmnat1.

## Methods

### Animal

All animal experiments were approved by the Animal Care Committee of the Institute of Medical Science, University of Tokyo, and conducted in accordance with the ARVO (Association for Research in Vision and Ophthalmology) statement for the use of animals in ophthalmic and vision research. For all embryonic mouse tissues analyzed, embryonic day (E) 0.5 was defined as the date vaginal plug was observed. Institute of Cancer Research (ICR) mice were obtained from Japan SLC Co., and we confirmed that the mice were free of Rd1 mutation. We used about 8-week-old mice when we indicate “adult mouse.”

### PCR and plasmids construction

Total RNA was purified from the mouse retina using Sepazol RNA I Super G (Nacalai Tesque) and cDNA was synthesized using ReverTra Ace qPCR RT Master Mix (TOYOBO). Quantitative PCR (qPCR) was performed using the SYBR Green-based method, using the Roche Light Cycler 96 (Roche Diagnostics). Glyceraldehyde 3-phosphate dehydrogenase (*Gapdh*) was used as an internal control. The primer sequences are shown in Supplementary Table 1. For the construction of shRNA expression plasmids, the target sequences (Supplementary Table 2) were determined by using siDirect (http://sidirect2.rnai.jp) or siRNA selection program at whitehead (http://sirna.wi.mit.edu). The shRNA plasmids were constructed as described previously^16^. As control, scramble sequences were used. To trace plasmid-transfected cells, we co-transfected an enhanced green fluorescent protein (EGFP)-expressing plasmid (pCAG-EGFP) with shRNA expression plasmid. shRNAs were prepared using at least two different target sequences for each gene and we observed essentially the same results between first and second shRNA. Representative data using shRNA first are shown in the figures.

### Electroporation and retinal explant culture

In vitro electroporation and retinal explant culture were performed as described previously^17,18^. Briefly, the total amount of plasmids used for electroporation was 100 μg for each retina and composition of plasmids in each experiment is shown in the list (Supplementary Table 4). Retinas dissected from E17.5 ICR mouse embryos were transferred to a micro-electroporation chamber filled with plasmid solution (1 mg/mL in Hanks’ balanced salt solution). Then, the retinas were electroporated with the plasmids and cultured as explants for 1–14 days as indicated in the text or legend to the figures according to the experimental condition. Then, electroporated retinas were placed on a Millicell chamber filter insert (Millipore) with outer nuclear layer (ONL) to the down upon the filter. The filters were placed into a six-well plate containing 1 ml of explant media, comprising 50% MEM-Hepes (Gibco), 25% Hank’s solution (−) (Gibco), and 25% heat-inactivated horse serum (JRH Biosciences) supplemented with 200 mM l-glutamine, 5.75 mg/ml glucose, and penicillin and streptomycin (Gibco), and cultured. For all the samples, we performed at least three independent electroporation with the same condition and counted cells from two or three sections in each sample. Then, average and SD were calculated. Statistical significance was calculated by Student’s *t*-test or one-way analysis of variance (ANOVA) followed by Tukey’s multiple-comparisons test according to experimental conditions. Details are shown in each figure legend.

### In vivo electroporation

In vivo electroporation was performed as previously described^19^. Briefly, plasmids shown in Supplementary Table 4 were injected into subretinal space of postnatal day (P) 0.5 mouse retinas and electric pulse was applied. Electroporated retinas were harvested after 14 days of breeding.

### Immunohistochemistry

Immunostaining of frozen sections was done as described previously^18,17^. Briefly, retinal explants were fixed with 4% paraformaldehyde and treated with 15% and 30% sucrose in this order, and embedded in the optimal cutting temperature compound (Sakura Fineteck). Primarily antibodies used were monoclonal antibodies against active Caspase 3 (Promega), Ki67 (BD Bioscience), HuC/D (Molecular Probes), Chx10 (Exalha Biologicals), NR2E3 (photoreceptor-specific nuclear receptor, PNR) (ppmx), glutamine synthetase (GS) (Millipore), PKC (Merck Millipore), Rhodopsin (Rho4D2, kindly donated by Dr R. S. Molday, The University of British Columbia), and a polyclonal antibody against GFP (Clontech). Nuclei were counterstained with 4′,6-diamidino-2-phnylindole, dihydrochloride (DAPI). Sections were then treated with Alexa-488-, Alexa-594-, or Alexa-680-conjugated appropriate secondary antibodies. Photos were taken under observation using Zeiss Axio Image M1 and Axio Image M2.

### TdT-mediated dUTP nick end labeling assay

TdT-mediated dUTP nick end labeling (TUNEL) assay was performed using In situ Apoptosis Detection Kit as per the manufacturer’s instructions (Takara Bio, Inc.). Retinal sections were prepared as described above. After staining, photographs were taken under Zeiss Axio Image M1 and Axio Image M2.

### In situ hybridization

In situ hybridization of Nmnat1 was performed using digoxigenin-labeled RNA probes as previously described^20^. Hybridization was performed at 55 °C for overnight. Probes used for in situ hybridization were cloned by reverse transcription (RT)-PCR using primers, Nmnat1_ISH_FW: 5′-gagggagtcagatcttgttg-3′, Nmnat1_ISH_RV: 5′-atcggtgagcctggctagag-3′. The fragment was inserted into pGEM-Teasy vector, and sense and antisense strands were synthesized by T7 RNA polymerase or SP6 RNA polymerase.

### ChIP-qPCR analysis

Chromatin immunoprecipitation (ChIP)-qPCR was done as previously described^21^. Sequences of qPCR to detect genomic region of *Fas* and *Noxa* loci are shown in Supplementary Table 3. The primers covers up to about − 800 bp from transcription initiation sites and the regions of interest were defined partly by referring to previous reports of human FAS and NOXA ChIP-qPCR^22,23^.

### Western blotting

Retinas were dissected from mice embryos at E17.5 and electroporated with sh-Scramble- or sh-Nmnat1-encoding plasmids. After 2 days of culture, cell lysates were collected from the retinal explants and used for western blotting. Primary antibodies against acetyl-Histone H3 (Millipore), acetyl-Histone H4 (Millipore), actin (Sigma), and horseradish peroxidase-linked secondary antibodies (GE Healthcare) were used.

### Measurement of NAD concentration

NAD content in mice explant retinas was measured using Amplite^TM^ Fluorimetric NAD/NADH Ratio Assay Kit (AAT Bioquest, Sunnyvale, CA, USA), according to the manufacturer’s instructions. Briefly, retinas dissected from E17.5 ICR mouse embryos were electroporated with the sh-Scramble or sh-Nmnat1, and cultured for 2 days. Then, the explant retinas were collected and measurement of NAD/NADH contents was performed in accord with the manufacturer’s instructions. The values of NAD content were divided by the area of explant retinas for normalization.

### Statistical analysis

The *p*-values were calculated by Student’s *T*-test or Tukey’s test as indicated in the figure legend.

## Results

### Expression patterns of Nmnat1 during retinal development

**Fig. 1:**
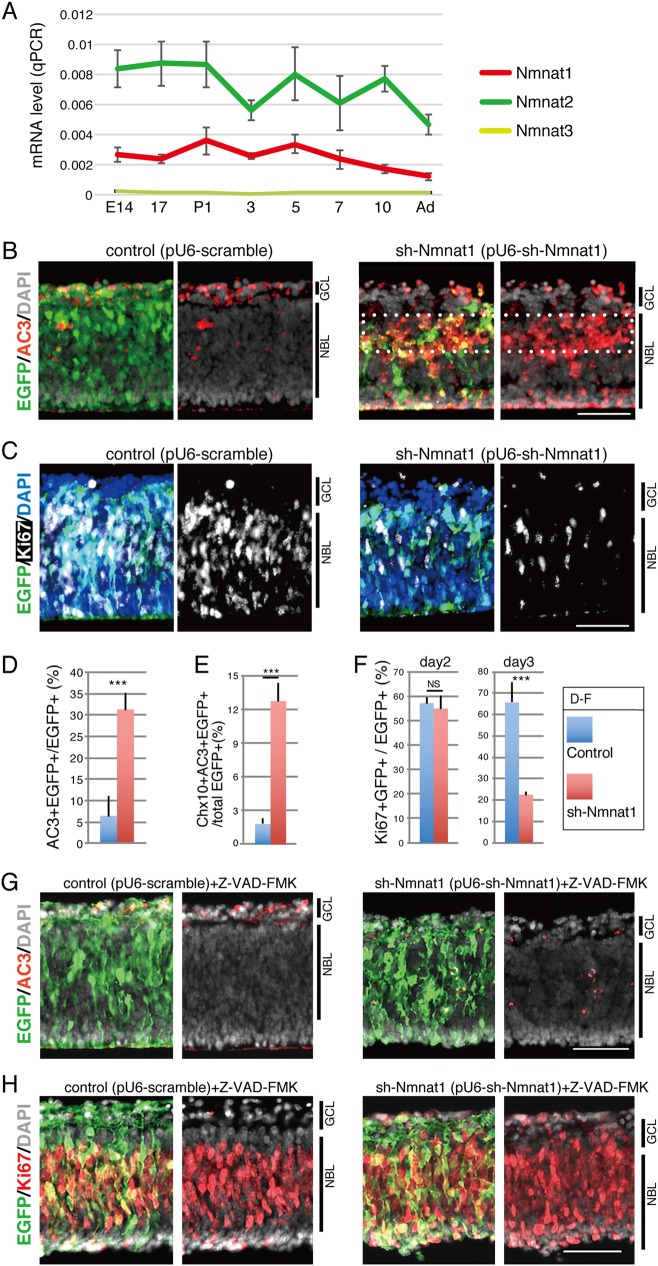
Effects of downregulation of Nmnat1 during retinal development lead to apoptosis. **a** Transition of the expression of mRNAs of Nmnat family members during mouse retinal development was examined by RT-qPCR. Whole retinas at indicated developmental stages were isolated, and RT-qPCR was performed. Relative expression levels to *Gapdh* are shown. The values are average of 3 independent samples with standard deviation. **b**, **c** Plasmids encoding sh-Nmnat1 or scramble control in combination with EGFP expression plasmid were electroporated into mouse isolated retina at E17.5, and the retinas were cultured for 3 or 4 days as explants and frozen sectioned. Immunostaining was done by using anti-active Caspase 3 (AC3) antibody to detect apoptosis (**b**) or anti-Ki67 antibody to detect proliferating cells (**c**). Transfected cells were stained with anti-GFP antibody, and nuclei were visualized by staining with DAPI (**b**, **c**). **d**–**f** Population of AC3 + EGFP + (**d**), Ki67 + EGFP + (**f**) cells in total EGFP-positive cells are shown. The sections at day 3 were stained with anti-Chx10 and -AC3 antibodies (**e**), with anti-GFP antibody (Supplementary Fig. 3C). Populations of triple-positive cells in total EGFP-positive cells are shown in **e**. The values are average of three independent samples with standard deviation. **g**, **h** Retinas at E17.5 were transfected with sh-Nmnat1 or control with EGFP expression plasmid and cultured for 3 days in the presence of Z-VAD-FMK at 20 μM in the final concentration. Frozen sections were stained with anti-AC3 (**g**) or -Ki67 (**h**) antibody with anti-GFP antibody. Nuclei were visualized by DAPI staining. Statistical significance was calculated by Student’s *T*-test. ***p < 0.005. Scale bar = 50 μm

First, the expression patterns of the Nmnat family of genes were examined in the developing mouse retina. Mouse retinas at the indicated developmental stages were isolated and the expression levels of *Nmnat1–3* transcripts were assessed by RT-qPCR (Fig. 1a). *Nmnat1* was expressed during the embryonic period and the expression levels declined slightly after postnatal day 5 (P5). *Nmnat2* was expressed in developing and mature retinas and its expression levels were relatively stable (Fig. 1a). The expression of *Nmnat3* was very low throughout the development of the retina (Fig. 1a). We then got information of more detailed expression patterns of *Nmnats* by examining retinal cell-type-specific RNA-seq (GSE71464), which was previously obtained in our laboratory^24^. *Nmnat1* was expressed both in Cd73-positive photoreceptor and Cd73-negative other retinal cell types (Supplementary Fig. 1A). Interestingly, *Nmnat2* was expressed in Cd73-negative cells but very weak in Cd73-positive cells, and *Nmnat3* expression was very low in both populations. To examine the spatial expression pattern of *Nmnat1* transcripts in the developing mouse retina, in situ hybridization was performed using digoxigenin-labeled RNA probes. *Nmnat1* was expressed relatively weak in the neuroblastic layer (NBL) and the ganglion cell layer (GCL) at embryonic day (E) E17 (Supplementary Fig. 1B, C). Its expression became higher in the inner side of NBL and GCL at P0. After the formation of three-layered structure of retina, its expression was observed in whole retinal layer but was relatively high in the inner nuclear layer (INL) and GCL at P7 and P14 (Supplementary Fig. 1B, C). In adult mouse retina, *Nmnat1* expression in GCL finally became very weak (Supplementary Fig. 1B, C).

### Expression of sh-Nmnat1 in the embryonic retina increased apoptosis and decreased proliferation in the developing retina

To examine the role of Nmnat1 during retinal development, loss-of-function analyses were performed in the developing retina using mouse retinal explant cultures. Plasmids carrying sh-Nmnat1 were constructed and shown to efficiently suppress *Nmnat1*, but not *Nmnat2*, in NIH3T3 cells (Supplementary Fig. 2A, B). Next, the plasmids encoding sh-Nmnat1 or a scramble control with an EGFP expression plasmid were transfected into isolated mouse retinas at E17.5 via electroporation. After 4 days of culture, the explants were cryosectioned and their phenotypes were examined with immunohistochemistry. The sh-Nmnat1-expressing retinas showed large numbers of active caspase 3 (AC3)-positive apoptotic cells in the inner side of the NBL and the EGFP-positive cells had mostly disappeared from this region (Fig. 1b, d). TUNEL assay on retinal explants cryosections also showed increased apoptosis in the NBL of sh-Nmnat1-expressing retina compared with that of control retina (Supplementary Fig. 3A). Chx10 is a marker of both retinal progenitor and bipolar cells depending on the retinal developmental stage. Chx10 was expressed in proliferating retinal progenitor cells in the retinal explants beginning on E17.5 and the explants were cultured for 3 days (Supplementary Fig. 3B, C). Co-immunostaining for anti-AC3, Chx10, the amacrine marker HuC/D, and the rod photoreceptor marker PNR revealed an increase in AC3-positive cells that was mostly evident in Chx10-positive progenitor cells (Fig. 1e and Supplementary Fig. 3D–G).

Cell proliferation was examined by expression of the proliferation marker Ki67. The number of Ki67-positive cells had not changed 2 days after the transfection of sh-Nmnat1 (Fig. 1f and Supplementary Fig. 4) but decreased dramatically after 3 days of culture (Fig. 1c, f). Next, whether the decrease in Ki67-positive cells in sh-Nmnat1-expressing retinas resulted from a reduction in progenitor cells due to apoptosis or a direct decrease in the proliferation following Nmnat1 depletion was evaluated. The administration of pan-caspase inhibitor Z-VAD-FMK (Adooq Bioscience) completely suppressed sh-Nmnat1-induced apoptosis (Fig. 1g), and the proliferation of sh-Nmnat1-expressing retinas was also rescued (Fig. 1h). These findings suggest that the suppression of proliferation was secondary to apoptosis in sh-Nmnat1-expressing retinas.

### Effects of Nmnat1 loss-of-function on differentiation in the retina

**Fig. 2:**
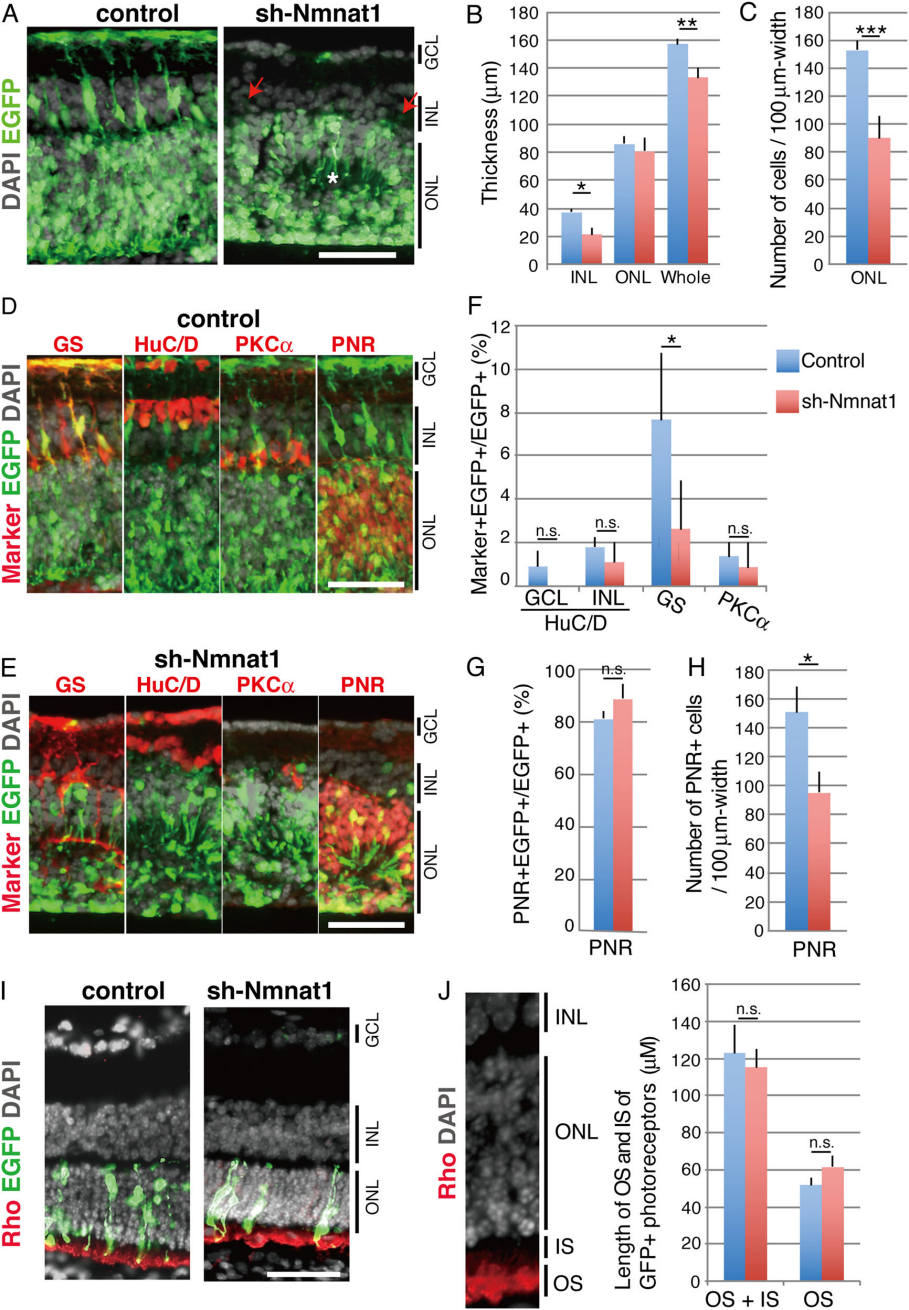
Retinal subtypes in INL were severely perturbed in sh-Nmnat1 expressing retina. **a**–**h** Retinas at E17.5 were transfected with scramble and sh-Nmnat1 and EGFP expression plasmids and cultured for 14 days. Frozen sections were stained with anti-GFP antibody (**a**) or anti-GFP antibody with retinal subtype markers (GS for Müller glia, HuC/D for amacrine, PKCa for bipolar, and PNR for rod photoreceptors) in **d** and **e**. Nuclei were visualized by DAPI staining. Thickness of INL, ONL, and whole retinas (**b**), the number of DAPI-positive cells per 100 μm in the ONL (**c**), and PNR-positive cells per 100 μm in the ONL (**h**) were measured. Marker + EGFP + double-positive cells in total EGFP cells are shown in **f** and **g**. The values are average of three independent samples with SD. Statistical significance was calculated by Student’s *T*-test. *p < 0.05, **p < 0.01, ***p < 0.005. Scale bar = 50 μm. **i**, **j** Mouse retinas at P0.5 were electroporated with scramble or sh-Nmnat1 with EGFP expression plasmids in vivo. After 14 days of breeding, retinas were collected and immunostaining with anti-rhodopsin antibody and anti-EGFP antibody. Nuclei was visualized with DAPI. **i**, **j** The region stained with anti-Rhodopsin antibody was defined as the outer segment (OS) and the space between the OS and the ONL was defined as the inner segment (IS) (**j**). The length of IS and OS of EGFP-positive photoreceptor was measured (**i**). The values are average of three independent samples with SD. Statistical significance was calculated by Student’s *T*-test. Scale bar = 50 μm

Next, the culture period of the retinal explants was extended to examine differentiation. At E17.5, the retinas were transfected with a scrambled control or sh-Nmnat1 plasmid with EGFP expression plasmids, cultured for 14 days, and the differentiation of retinal subtypes was examined with immunohistochemistry. After 14 days, the retinal explants formed three-layered structures but the INL in the sh-Nmnat1-expressing retinas was much thinner than in the controls (Fig. 2a, b). In addition, very few EGFP-positive cells were observed in the INL of sh-Nmnat1-expressing retinas (Fig. 2a, arrows). In the ONL of the explants, rosette-like structures were observed in sh-Nmnat1-expressing retinas (Fig. 2a, asterisk), but the thickness was only slightly thinner than that of the controls (Fig. 2b). To confirm the effect of sh-Nmnat1 on the ONL in more detail, the number of cells in the ONL was compared between scramble- and sh-Nmnat1-expressing retinas. The number of cells per 100 μm was significantly decreased in sh-Nmnat1-expressing retina compared with that of control (Fig. 2c).

The morphology of GS-positive Müller glial cells was severely perturbed and the number of EGFP- and GS-double-positive cells was significantly lower in the sh-Nmnat1-expressing retinas (Fig. 2d–f). However, number and alignment of HuC/D-positive amacrine cells were comparable between the control and sh-Nmnat1-transfected cells (Fig. 2d–f). HuC/D-positive displaced amacrine cells in GCL were also not significantly different between control and sh-Nmnat1-expressing retina (Fig. 2d–f). The number of PKCα-positive rod bipolar cells did not significantly differ between the control and sh-Nmnat1-expressing retinas (Fig. 2f). PNR-positive rod photoreceptors were located in the ONL and comparable numbers of EGFP- and PNR-double-positive cells were apparent between the sh-scramble- and sh-Nmnat1-expressing retinas (Fig. 2d, e, g). However, when we examined the exact number of cells (Fig. 2c) and PNR-positive cells (Fig. 2h) in ONL, both were significantly lower in sh-Nmnat1-expressing retina compared with that of control (Fig. 2h).

To examine the effect of sh-Nmnat1 on the formation of photoreceptor inner and outer segments (IS and OS), in vivo electroporation was performed. Scramble- or sh-Nmnat1 plasmid were injected into subretinal space of P0.5 mouse eyes and then were electroporated into mouse retina. After 14 days of breeding, length of IS and LS was compared. The region stained with anti-rhodopsin antibody was defined as the OS, and the region between the ONL and the OS was regarded as the IS (Fig. 2j). Length of OS and IS of EGFP-positive photoreceptors was comparable between control and sh-Nmnat1-expressing retinas (Fig. 2i, j).

### Expression of sh-Nmnat1 led to apoptosis in the NBL of the post-mitotic retina

Next, the effect of Nmnat1 downregulation was examined in later stages of retinal development. At P0.5, retinas were isolated and electroporated with sh-Nmnat1 and the explants were cultured for 4 days. The anti-AC3 antibody staining revealed several apoptotic cells in the NBL; however, the population was much smaller compared with retinas in which sh-Nmnat1 was introduced at E17.5 (Supplementary Fig. 5A, B). After 10 days of culture, immunostaining for other retinal subtype markers revealed the perturbed expression of GS, a marker of Müller glial cells (Supplementary Fig. 5C).

### Addition of NAD reversed the Nmnat1 loss-of-function-induced phenotype

**Fig. 3:**
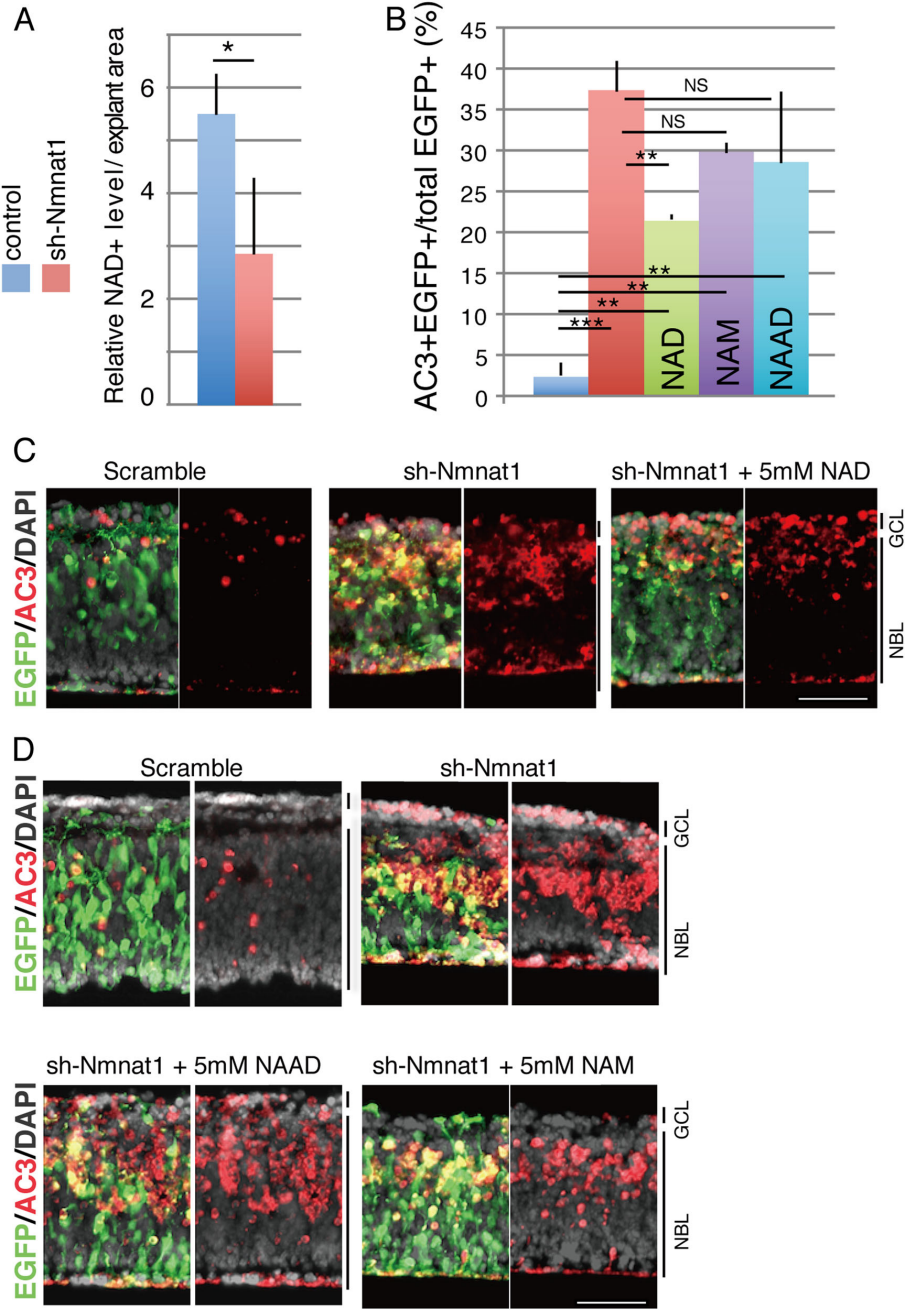
Addition of NAD and its derivatives into the retinal culture expressing sh-Nmnat1 modulated apoptotic cells from inner side of neuroblastic layer. **a** Levels of NAD in the retina expressing sh-Nmnat1 or scrambled control was examined by colorimetric method. After 2 days of culture, whole retinas were harvested, and NAD contents were examined. The values are average of three independent samples with SD. Statistical significance was calculated by Student’s *T*-test. *p < 0.05. **b**–**d** Isolated retinas at E17.5 were electroporated with the plasmids encoding sh-Nmnat1 or scrambled control in combination with EGFP expression plasmid and cultured in the presence of NAD (**c**), NAAD, or NAM (**d**) at indicated final concentration in the media. After 4 days of the culture, retinas were frozen sectioned, and immunohistochemistry was done using anti-AC3 and -EGFP antibodies. The values are average of three independent samples with SD. Statistical significance was calculated by one-way ANOVA followed by Tukey’s multiple-comparisons test. **p < 0.01, ***p < 0.005. Scale bar = 50 μm

As Nmnat1 is an essential enzyme in the NAD biosynthesis pathways, the present study assessed whether NAD content changed in sh-Nmnat1-expressing cells. Intracellular NAD levels were measured using a colorimetric method, which revealed decreased levels of NAD after the expression of sh-Nmnat1 (Fig. 3a). Next, the effects of extracellular supplementation of NAD on sh-Nmnat1-induced apoptosis were assessed. Connexin-43 (Cx43, Gja1) is a plasma membrane NAD transporter^25^ and the expression of *Cx43* was confirmed in both rod photoreceptors and other cells in the developing and mature retina using RNA-Seq data (Supplementary Fig. 6A). NAD was added to the culture medium of retinas transfected with control or sh-Nmnat1 with the EGFP expression plasmid at a final concentration of 5 mM. On day 4 of culture, NAD (5 mM) inhibited the appearance of AC3-positive cells on the inner side of the NBL (Fig. 3b, c). However, 1 and 10 mM NAD did not rescue the phenotype (Supplementary Fig. 6B), which suggests that an appropriate concentration of NAD is important for this rescue. Next, the effects of NAM and nicotinic acid adenine dinucleotide (NAAD), which are both involved in the NAD synthesis pathways (Supplementary Fig. 6C), were examined. The addition of either NAM or NAAD slightly lowered the number of AC3 apoptotic cells but these differences were not statistically significant (Fig. 3b, d).

### Sirt1 and Sirt6 are involved in early retinal development

**Fig. 4:**
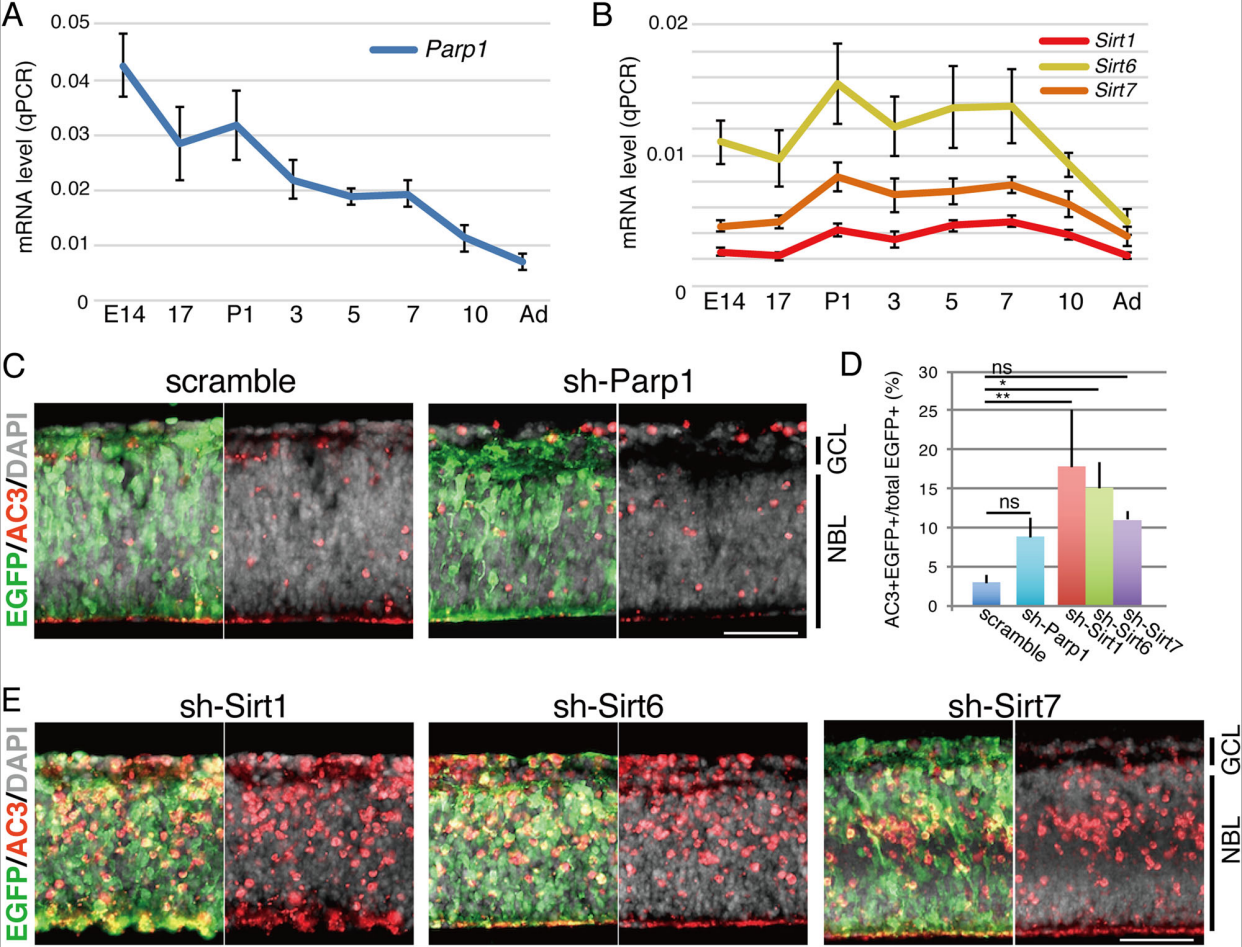
Effects of downregulation of Parp1, Sir1, 6, or 7 in retinal development. **a**, **b** Time course of expression of *Parp1* (**a**), *Sirt1, Sirt6,* and *Sirt7* (**d**) transcripts was examined by RT-qPCR using whole retina at indicated developmental stages. Relative expression levels to *Gapdh* are shown. Three independent samples were examined, and results with SD are shown. **c**–**e** Plasmids encoding sh-Parp1 (**c**), -Sirt1, -Sirt6, or -Sirt7 (**e**), or scrambled control in combination with EGFP expression plasmid were electroporated into mouse isolated retina at E17.5 and the retinas were cultured for 4 days as explants. Cryosections were stained with anti-AC3 antibody to detect apoptotic cells and anti-EGFP antibody to visualize transfected cells. Nuclei were stained with DAPI (gray). AC3 + EGFP + double-positive cells in total EGFP + cells were calculated **d**. Values are average of three independent transfected cells with SD. Statistical calculation was done by one-way ANOVA followed by Tukey’s multiple-comparison test. *p < 0.05, **p < 0.01. Scale bar = 50 μm

As decreased levels of NAD mediated the phenotype induced by sh-Nmnat1 expression in the retina, the roles of NAD-dependent nuclear enzymes necessary for retinal development were investigated. In particular, we focused on poly ADP-ribose polymerase 1 (Parp1) and Sirtuins (Sirts) as candidates involved in the sh-Nmnat1-induced phenotype. Parp1 catalyzes the poly-adenosine diphosphate (ADP) ribosylation of proteins involved in various physiological processes^26^. RT-qPCR analysis revealed that the expression of *Parp1* decreased consistently during retinal development (Fig. 4a) Sirt1, Sirt6, and Sirt7 are localized to the nucleus in most cell types^27,28^, and qPCR analysis revealed that these molecules were expressed in the developing retina at relatively constant levels (Fig. 4b).

Plasmids encoding sh-Parp1, -Sirt1, -Sirt6, or -Sirt7 were constructed and their ability to suppress their respective targets was examined in NIH3T3 cells using RT-qPCR (Supplementary Fig. 7A–D). At E17.5, isolated retinas were electroporated with one of the shRNA expression plasmids (sh-Parp1, -Sirt1, -Sirt6, or -Sirt7) together with the EGFP expression plasmid and cultured for 4 days. Immunostaining with anti-AC3 revealed that sh-Parp1 slightly enhanced the number of AC3-positive cells, although not significantly (Fig. 4c, d). In contrast, the expression of sh-Sirt1 and sh-Sirt6 showed strong AC3-positive signals (Fig. 4d, e); sh-Sirt7 also increased the number of AC3-positive cells but this difference was not statistically significant (Fig. 4d, e). AC3-positive cells were mainly localized in the inner half of the NBL and RGC in sh-Sirt1- and sh-Sirt6-expressing retinas, similar to the phenotype observed in the sh-Nmnat1-expressing retinal explants (Fig. 4e).

### Upregulation of Noxa and Fas during Nmnat1 depletion-induced apoptosis in the retina

**Fig. 5:**
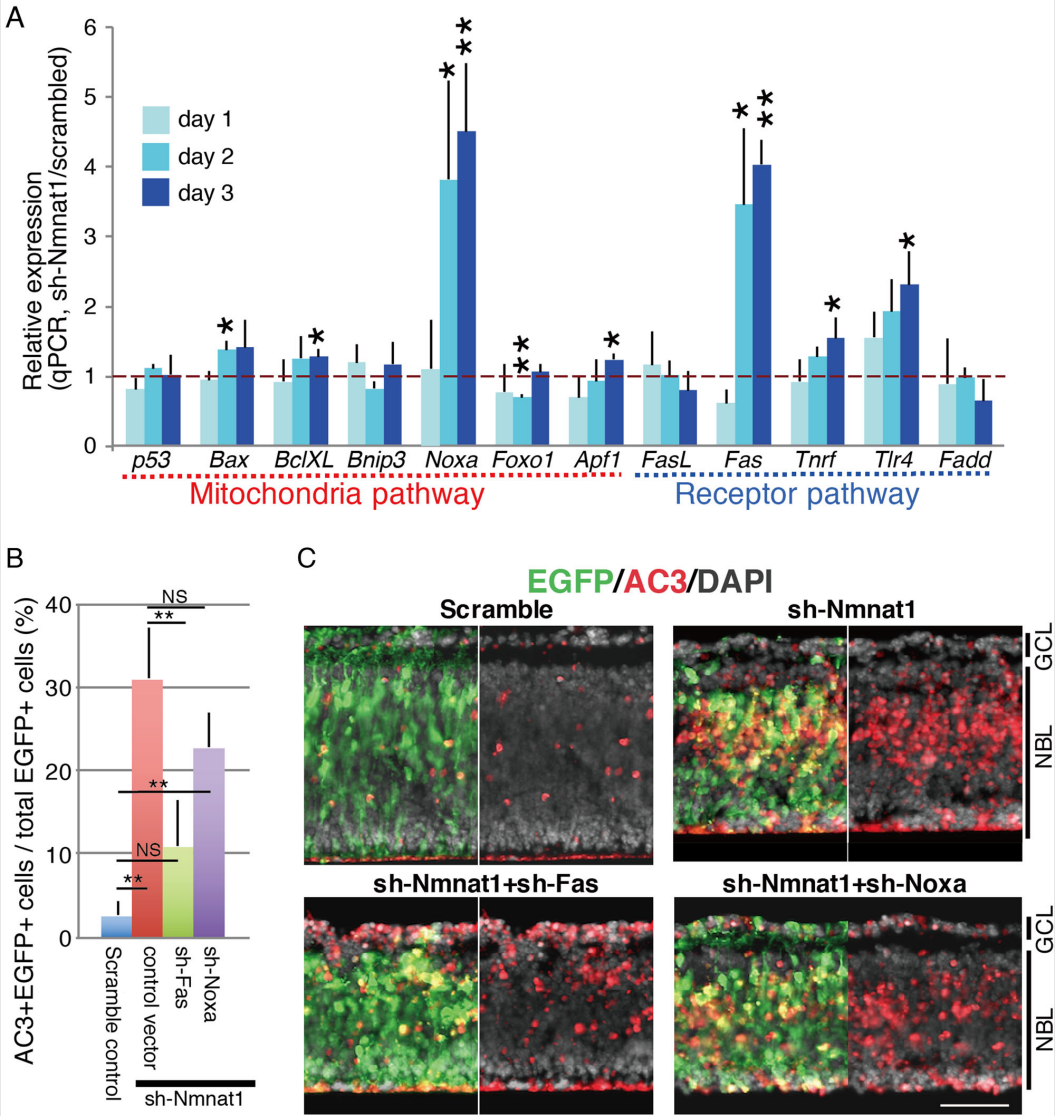
Expression of apoptosis related genes in retina expressing sh-Nmnat1. **a** The retinas at E17.5 were electroporated with plasmids encoding sh-Nmnat1 or scrambled control in combination with EGFP expression plasmid and cultured as explant. Then the retinas were harvested at 1, 2 and 3 days of the culture, and RT-qPCR to detect pro-apoptotic related genes was done. Values are relative to control *Gapdh* value and average of three independent transfected samples with standard deviation. Statistical calculation was done by Student’s *T*-test. *p < 0.05, **p < 0.01. **b**, **c** Examination of effects of sh-Fas or sh-Noxa. Retinas at E17.5 were transfected with control or sh-Nmnat1 together with sh-Fas or sh-Noxa, and cultured for 4 days. Apoptosis was examined by anti-AC3 and –EGFP antibodies (**c**), and AC3 + EGFP + cells in total EGFP-positive cells are shown in **b**. Values are average of three independent transfected samples with SD. Statistical analysis was done one-way ANOVA followed by Tukey’s multiple-comparison test. **p < 0.01. Scale bar = 50 μm

To identify the genes responsible for the induction of apoptosis following Nmnat1 depletion, the expression levels of pro-apoptotic genes were examined. At E17.5, isolated retinas were electroporated with scramble control or sh-Nmnat1 plasmids and collected after 1, 2, or 3 days of culture. RT-qPCR analysis of various pro-apoptotic genes revealed that *Noxa* and *Fas* were strongly induced by sh-Nmnat1 after 2 and 3 days of culture (Fig. 5a). Various other genes, such as *Bax*, *BclXL*, *Tnrf*, *Tlr4*, and *Apf1*, exhibited weak increases in expression (Fig. 5a).

To determine whether *Noxa*, *Fas*, or both were involved in sh-Nmnat1-induced apoptosis in the retina, shRNA molecules targeting *Fas* and *Noxa* were constructed and the degree of efficiency of the shRNA constructs was examined in NIH3T3 cells (Supplementary Fig. 7E and 7F). At E17.5, the retinas were co-transfected with sh-Nmnat1- and sh-Fas- or sh-Noxa-expressing plasmids. After 4 days of culture, sh-Fas significantly reduced the number of apoptotic cells (Fig. 5b, c). Although sh-Noxa also reduced the number of apoptotic cells, this difference was not statistically significant (Fig. 5b, c).

### Increased acetylation of histones H3 and H4 in sh-Nmnat1-expressing retinas

**Fig. 6:**
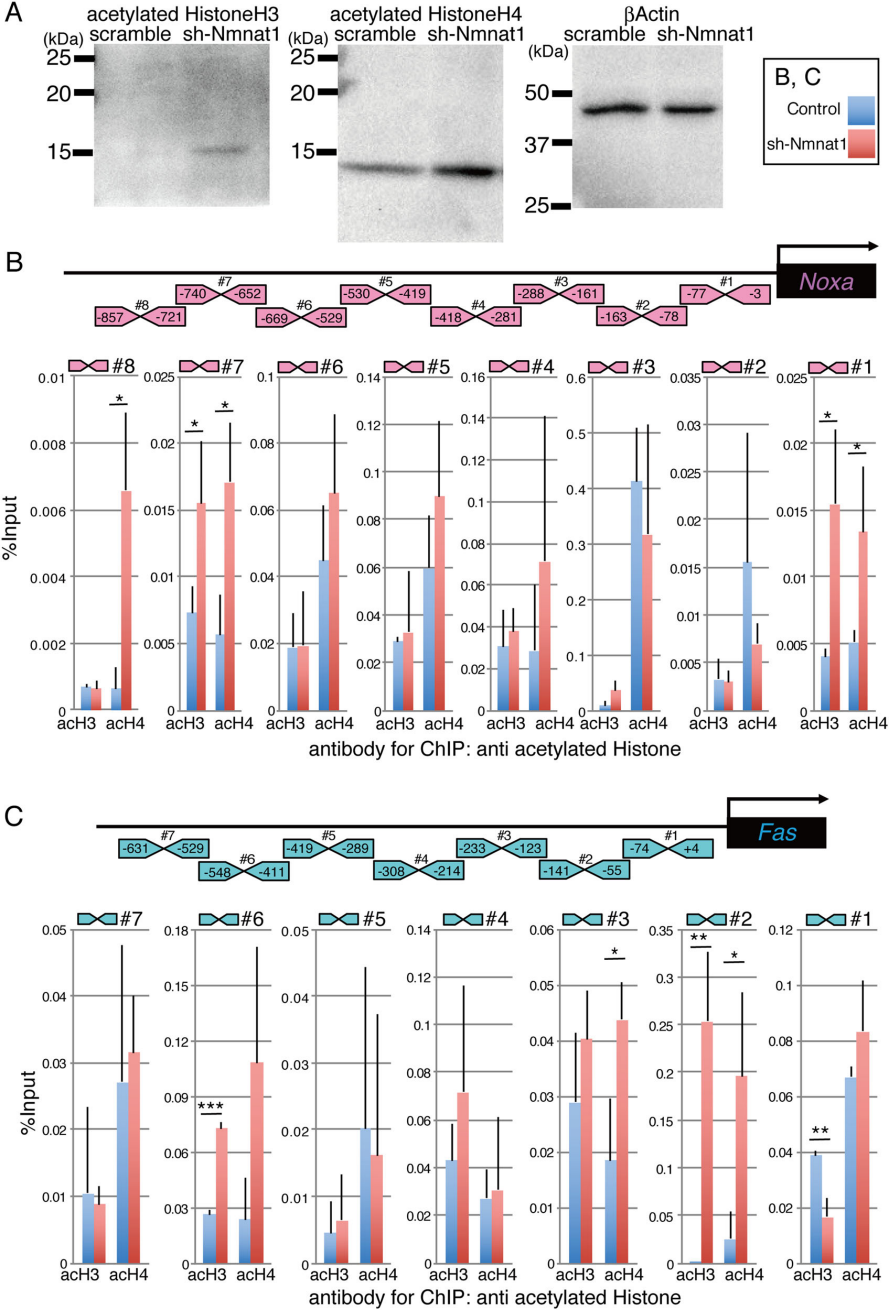
Histone H3 and Histone H4 acetylation levels were enhanced in Nmnat1-knockdown retina. **a** Plasmid encoding sh-Nmnat1 or scrambled control were transfected into retina at E17.5 and cultured for 2 days, and total proteins were harvested. Western blotting using anti-acetylated Histone H3, -acetylated Histone H4, or -beta-actin antibodies was done. The experiments were repeated three times, and essentially the same results were obtained. **b**, **c** Schematic representation of 5′-non-coding region of *Noxa* and *Fas* gene loci. Region of primers used for Chip-qPCR are shown (Noxa_#1-7, Fas_#1-8). **c** Isolated retinas at E17.5 were transfected with control or sh-Nmnat1 and retinas were collected after 2 days. Chip using anti-acetylated HistoneH3 or -acetylated HistoneH4 were performed, and qPCR using primers shown in **b** and **c** was done. Values are % input and average of three independent transfected samples with SD. Statistical analysis was done by Student’s *T*-test. *p < 0.05, **p < 0.01, ***p < 0.005

As Sirts are histone H3 and H4 deacetylases^29^, it was hypothesized that the sh-Nmant1-induced decrease in NAD would lead to Sirt dysfunction and, as a result, the acetylation of histones H3 and H4 would increase. To test this hypothesis, sh-Nmnat1 was transfected into isolated retinas at E17.5 and the acetylation of histones H3 and H4 was examined by western blotting after 2 days of culture. Histone H3 and H4 acetylation increased in sh-Nmnat1-expressing retinas (Fig. 6a). Subsequently, ChIP-qPCR using anti-acetylated histone H3 or H4 antibodies and primers designed for the indicated positions of the *Noxa* and *Fas* gene loci (Fig. 6b, c) was performed. The acetylation levels of histones H3 and H4 in these regions were upregulated by the expression of sh-Nmnat1 (Fig. 6b, c).

## Discussion

The present study found that Nmnat1 is essential for the early development of the retina, particularly for the survival of retinal progenitor cells that are fated to retinal subtypes in the INL. This result was unexpected, because it was hypothesized that Nmnat1 is involved in the development and/or maintenance of photoreceptors, not in other retinal subsets or progenitors in terms of the LCA pathological phenotype.

Recent studies using NAD sensors have reported that the depletion of Nmnat1 results in less cytoplasmic NAD in cells expressing Nmnat2 at a low level but not in Nmnat2-rich cells^30^. Nmnat1 knockout is lethal in mouse embryos^14^, which supports the idea that extranuclear Nmnat isoforms cannot compensate for Nmnat1 activity, at least during embryonic development. Although Nmnat2 was abundantly expressed in retinal progenitors, it is possible that the maintenance of nuclear NAD levels by Nmnat1 is essential for the survival of retinal progenitor cells, and its fail is primary reason of progenitor apoptosis.

In the present study, the concentration of NAD was reduced in whole cell extracts from sh-Nmnat1-expressing retinas. This decrease was slight but expected, because the concentration of NAD in the cytoplasm may not be affected by the presence of Nmnat2. The addition of NAD reversed the apoptotic phenotype induced by the depletion of Nmnat1. Cx43 transports NAD from extracellular areas to intracellular areas^25^ and the expression of *Cx43* transcripts in the developing retina was confirmed by our RNA-Seq analysis. However, the transport of NAD from the cytoplasm to the nucleus has yet to be confirmed and may raise some skepticism, because Nmnat2 did not compensate for Nmnat1 depletion in the retinal progenitors. It is notable that excess amounts of NAD could not rescue the phenotype, and that the co-expression of Nmnat1 with sh-Nmnat1 did not rescue the phenotype in the present study (data not shown). These results suggest that an excess supply of NAD may be toxic in the retina. Although the toxicity of the excess administration of NAD to mice had been known, mechanisms of the toxicity of NAD had not been well understood.

Mice with mutated Nmnat1 can be found in the chemical mutagenesis mouse pool and it has been shown that mice with a homozygous *Nmnat1* mutation develop a rapidly progressing chorioretinal disease that begins with photoreceptor degeneration^15^. Reduced retinal vasculature, optic atrophy, and retinal pigment epithelium were reported, suggesting that photoreceptors are most vulnerable to disruptions in NMNAT1, because they are the first cell type to show signs of disease at 1 month in the mutant mice^15^. Therefore, wild-type Nmnat1 is also essential for photoreceptor maintenance in mice. However, as in LCA patients, the mutation described in the mouse model was not caused by malformations during the development of the retina, which differs from the observations made following Nmnat1 depletion in the present study. Therefore, it is plausible that another function of Nmnat1, distinct from its enzymatic influence on the NAD synthesis pathways, plays a pivotal role in rod survival. On the other hand, a detailed examination of the structural development of each cell type in the INL was not described in that study and, similarly, detailed information on LCA patients is not available. Based on the present findings, it is not appropriate to exclude the possibility that the incomplete differentiation of the INL contributes to the phenotype. Therefore, detailed examination of retinal structures with Nmnat1 mutations will be important.

The present data also revealed the roles of Sirt1 and Sirt6 in the protection of retinal progenitors from apoptosis. The importance of Sirt6 in retinal function is likely exerted through the control of the histone H3K9 and H3K56 acetylation^31^. Decreased levels of Sirt1 have been shown in the ONL of Rd10 mice^32^, and other Sirts have also been implicated in retinal diseases^33^. However, the role of Sirts with respect to retinal progenitor cells has not been well studied. In the present study, we showed a specific de-suppression of the expression of *Fas* and *Noxa*, both of which are pro-apoptotic genes, which likely resulted from an increase in the acetylation of histones H3 and H4 in the 5′-untranslated region of these genes. The acetylation of histones H3 and/or H4 in human *NOXA* and *FAS* promoters has been reported^22,23^. These findings suggest that the regulation of histone de-acetylation in the 5′-genomic region of *Noxa* and *Fas* is critical to avoid the unfavorable expression of pro-apoptotic genes in various tissues. Many studies have investigated the anti-apoptotic activity of Sirts and their involvement in the p53 and nuclear factor-κB pathways^34,35^; many other mechanisms have been suggested as well. In the present study, the protein stability of p53 in retinas expressing sh-Nmnat1 was examined but the results were not conclusive (data not shown).

The importance of NAD for the maintenance of photoreceptors has been clearly shown in a recent report that investigated mice with rod- or cone-specific deletion of NAM phosphorybosyltransferase (Nampt), which is the rate-limiting enzyme in the major NAD + biosynthetic pathway^36^. Analyses of these mice revealed retinal degeneration that could be rescued by the addition of NMN^36^. These authors also reported dysfunction of the mitochondrial deacetylases Sirt3 and Sirt5, which are important for the maintenance of NAD homeostasis. These findings suggest the importance of maintaining NAD concentration as a master regulator of photoreceptor metabolism^36^. Deficiencies in Nampt may led to decreased levels of NAD in whole cells, which is different from condition in LCA patients and our sh-Nmnat1-expressing retinas. However, the low level of Nmnat2 in the rod photoreceptors indicates that the cytoplasmic level of NAD was also affected by Nmnat1 mutations. To clarify this question, careful examination of subcellular NAD homeostasis should be conducted using different combinations of Nmnat1 mutations in rod photoreceptors.

## Electronic supplementary material

Supplemental figures

Supplemental Tables

## References

[1] Leber T (1869). Ueber retinitis pigmentosa und angeborene amaurose. Albrecht Von. Graefes Arch. Ophthal..

[2] Alkharashi M, Fulton AB (2017). Available evidence on leber congenital amaurosis and gene therapy. Semin. Ophthalmol..

[3] Chacon-Camacho OF, Zenteno JC (2015). Review and update on the molecular basis of Leber congenital amaurosis. World J. Clin. Cases..

[4] Wang J (2012). Exome sequencing identifies NMNAT1 mutations as a cause of Leber congenital amaurosis. Nat. Genet..

[5] Falk M (2012). NMNAT1 mutations cause Leber congenital amaurosis. Nat. Genet..

[6] Koenekoop R (2012). Mutations in NMNAT1 cause Leber congenital amaurosis and identify a new disease pathway for retinal degeneration. Nat. Genet..

[7] Perrault I (2012). Mutations in NMNAT1 cause Leber congenital amaurosis with early-onset severe macular and optic atrophy. Nat. Genet..

[8] Lau C, Niere M, Ziegler M (2009). The NMN/NaMN adenylyltransferase (NMNAT) protein family. Front. Biosci..

[9] Berger F, Lau C, Dahlmann M, Ziegler M (2005). Subcellular compartmentation and differential catalytic properties of the three human nicotinamide mononucleotide adenylyltransferase isoforms. J. Biol. Chem..

[10] Deng Y (2015). A novel missense NMNAT1 mutation identified in a consanguineous family with Leber congenital amaurosis by targeted next generation sequencing. Gene.

[11] Siemiatkowska AM (2014). Novel compound heterozygous NMNAT1 variants associated with Leber congenital amaurosis. Mol. Vis..

[12] Coppieters F (2015). Hidden genetic variation in LCA9-associated congenital blindness explained by 5’UTR mutations and copy-number variations of NMNAT1. Hum. Mutat..

[13] Coleman MP, Freeman MR (2010). Wallerian degeneration, WldS, and Nmnat. Annu. Rev. Neurosci..

[14] Conforti L (2011). Reducing expression of NAD + synthesizing enzyme NMNAT1 does not affect the rate of Wallerian degeneration. Febs. J..

[15] Greenwald S (2016). Mouse models of NMNAT1-Leber congenital amaurosis (LCA9) recapitulate key features of the human disease. Am. J. Pathol..

[16] Satoh S (2009). The spatial patterning of mouse cone opsin expression is regulated by bone morphogenetic protein signaling through downstream effector COUP-TF nuclear receptors. J. Neurosci..

[17] Tabata Y (2004). Retinal fate specification of mouse embryonic stem cells by ectopic expression of Rx/rax, a homeobox gene. Mol. Cell. Biol..

[18] Iida A, Shinoe T, Baba Y, Mano H, Watanabe S (2011). Dicer plays essential roles for retinal development by regulation of survival and differentiation. Invest. Ophthalmol. Vis. Sci..

[19] Matsuda T, Cepko CL (2004). Electroporation and RNA interference in the rodent retina in vivo and in vitro. Proc. . Natl Acad. Sci. USA.

[20] Koso H, Satoh S, Watanabe S (2007). c-kit marks late retinal progenitor cells and regulates their differentiation in developing mouse retina. Dev. Biol..

[21] Iida A (2014). Histone demethylase Jmjd3 is required for the development of subsets of retinal bipolar cells. Proc. Natl Acad. Sci. USA.

[22] Huang SK (2013). Histone modifications are responsible for decreased Fas expression and apoptosis resistance in fibrotic lung fibroblasts. Cell Death Dis..

[23] Wirth M (2014). Myc and egr1 synergize to trigger tumor cell death by controlling noxa and bim transcription upon treatment with the proteasome inhibitor bortezomib. Nucleic Acids Res..

[24] Ueno K (2016). Transition of differential histone H3 methylation in photoreceptors and other retinal cells during retinal differentiation. Sci. Rep..

[25] Song EK (2011). Connexin-43 hemichannels mediate cyclic ADP-ribose generation and its Ca2-mobilizing activity by NAD/Cyclic ADP-ribose transport. J. Biol. Chem..

[26] Krishnakumar R, Kraus WL (2010). The PARP side of the nucleus: Molecular actions, physiological outcomes, and clinical targets. Mol. Cell.

[27] Chalkiadaki A, guarente L (2012). Sirtuins mediate mammalian metabolic responses to nutrient availability. Nat. Rev. Endocrinol..

[28] Guarente L (2013). Calorie restriction and sirtuins revisited. Genes & Dev..

[29] Imai SI, Guarente L (2010). Ten years of NAD-dependent SIR2 family deacetylases; implications for metabolic diseases. Trends Pharmacol. Sci..

[30] Cambronne XA (2016). Biosensor reveals multiple sources for mitochondrial NAD+. Science.

[31] Silberman DM (2014). SIRT6 is required fo normal retinal function. PLoS ONE.

[32] Jaliffa C (2009). Sirt1 involvement in rd1- mouse retinal degeneration. IOVS.

[33] Balaiya S, Abu-Amero KK, KOndkar AA, Chalam KV (2017). Sirtuiins expression and their role in retinal diseases. Oxid. Med. Cell Longev..

[34] Imai SI, Guarente L (2014). NAD + and sirtuins in aging and disease. Trends Cell Biol..

[35] Watroba M (2017). Sirtuins, epigenetics and longevity. Ageing Res. Rev..

[36] Lin JB (2016). NAMPT-mediated NAD + biosynthesis is essential for vision in mice. Cell Rep..

