# Supplementary material for: Roles of Nmnat1 in the survival of retinal progenitors through the regulation of pro-apoptotic gene expression via histone acetylation
Source: Cell Death Dis. 2018 Aug 30;9(9):891. doi: 10.1038/s41419-018-0907-0 (PMC6117278; doi:10.1038/s41419-018-0907-0)
Supplement: Supplementary file 1 — Supplemental figures [file 41419_2018_907_MOESM1_ESM.pdf]

Supplemental Figure1

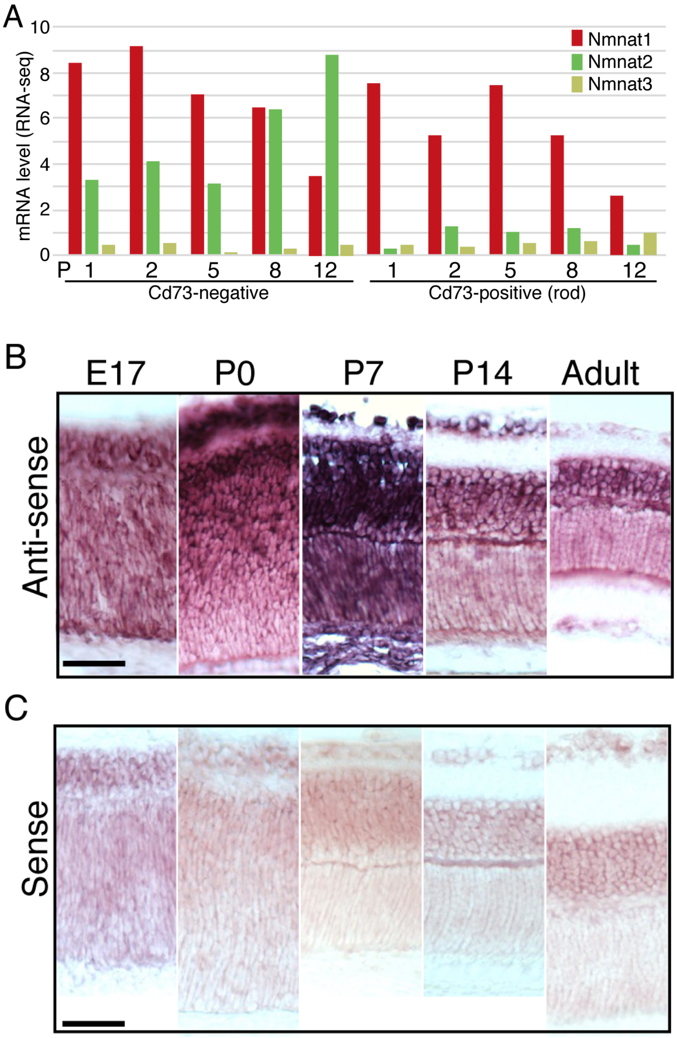

A. The expression levels of *Nmnat1, 2,* and *3* transcripts in developing photoreceptors and other retinal cells were examined in RNA-seq (GSE71464). Mouse retinas were isolated and fractionated to Cd73 positive photoreceptor and C73 negative other retinal cells, and RNA-seq was performed using total RNA purified from the fractions (Ueno et al., Sci Rep 6, 29264, 2016). FPKM values of *Nmnat1, 2, 3* are shown. B, C. In situ hybridization of *Nmnat1* using retinal sections at indicated developmental stages. Anti-sense (B) and sense (C) probes were used.

Supplemental Figure 2

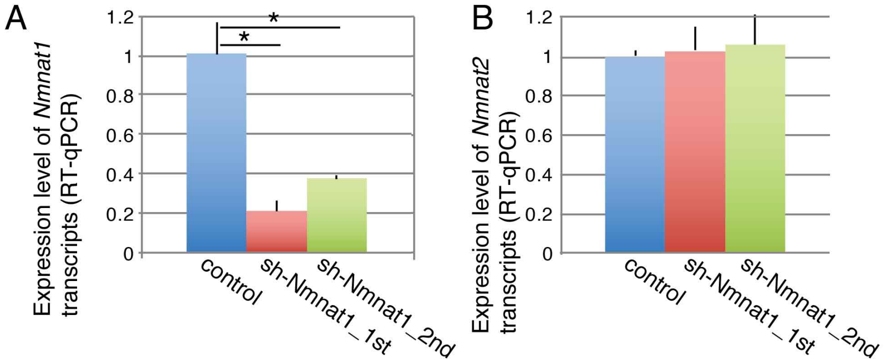

A, B. Plasmids containing scramble, sh-Nmnat1_1, or sh-Nmnat1_2 was transfected into NIH3T3 cells, and the cells were harvested after 48 hours of culture. RT-qPCR to detect *Nmnat1* (A), and *Nmnat2* (B) transcripts was done. Values are expressed relative to values of *Gapdh*. Three independent transfection was done, and average with standard deviation was performed. Statistical calculation was performed by Turkey HSD, p, $*<0.05$.

Supplemental Figure 3

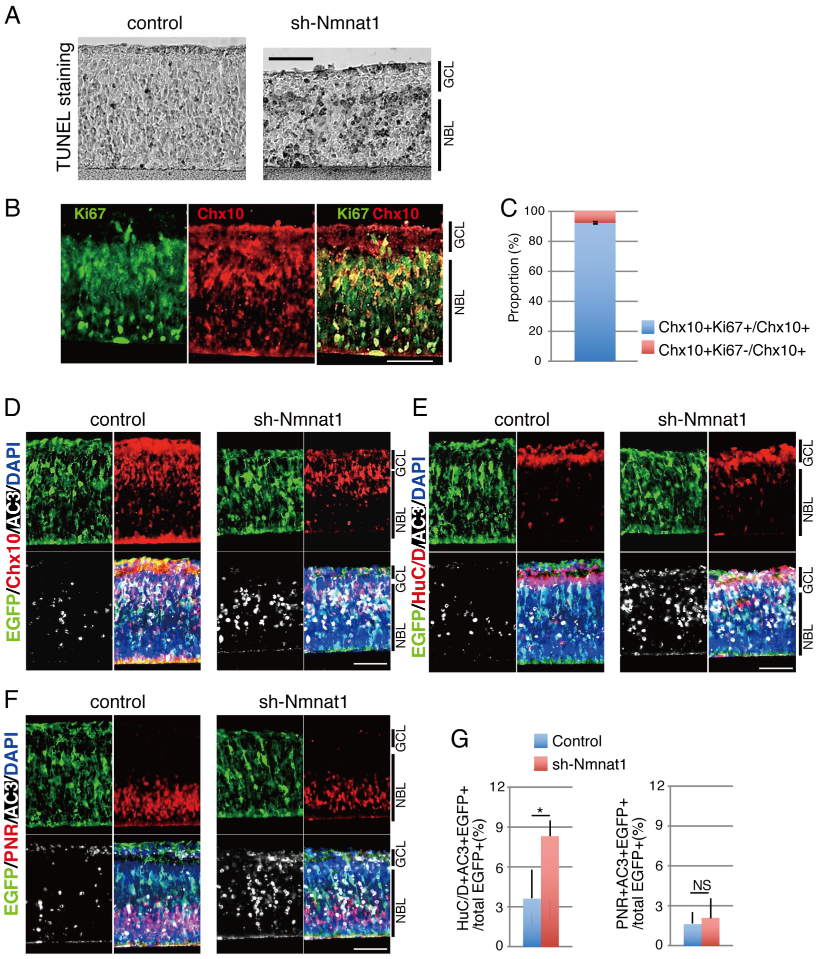

A. Isolated retinas at E17.5 were transfected with control or sh-Nmnat1 plasmid with EGFP-expressing plasmid, and after 4 days, TUNEL was done to detect DNA fragments. B, C. Mice retinas were isolated at E17.5, and immunostaining was performed after 3 days of culture by using anti-Ki67 and -Chx10 antibodies. Population of Chx10+Ki67+ double positive cell or Chx10+Ki67- single positive cells in total Chx10 positive cells was calculated in C. Values are average of three independent samples with standard deviation. D-G. Isolated retinas at E17.5 were transfected with control or sh-Nmnat1 plasmid with EGFP-expressing plasmid, and after 4 days, cryosections were immunostained with anti-EGFP, -AC3 antibodies with either one of anti-Chx10 (D), -HuC/D (E), or –PNR antibodies (F). Nuclei were visualized by staining with DAPI. Population of HuC/D+AC3+EGFP+ triple positive cells (left panel) or PNR+AC3+EGFP+ triple positive cells (right panel) in total EGFP+ cells are shown in G. Transfection was performed three times independently (D-F), and average with standard deviation are shown in F. Statistical calculation was performed by Student's T-Test , p, $*<0.05$, n.s.>0.05. Scale bar = 50 μm.

Supplemental Figure 4

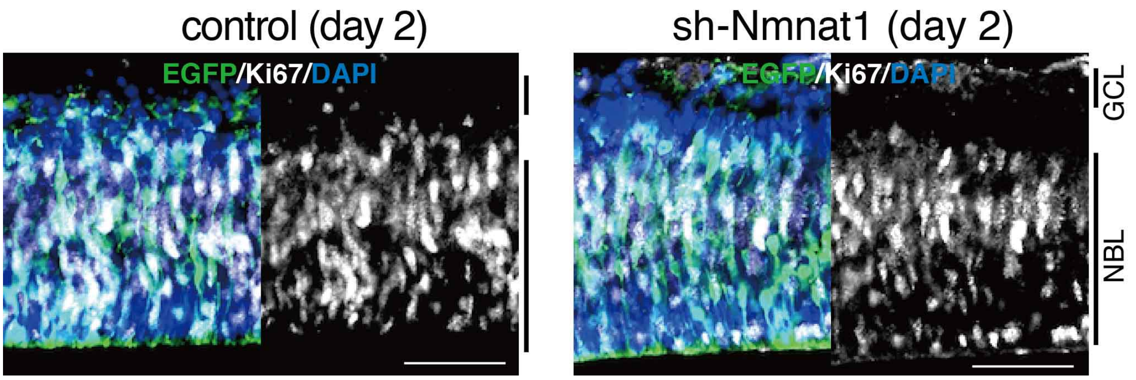

Cell proliferation was examined by anti-Ki67 antibody after 2 days of retinal explant culture transfected with control or sh-Nmnat1 together with EGFP expression plasmid at E17.5. Nuclei were visualized by staining with DAPI. Scale bar = 50 μm.

Supplemental Figure 5

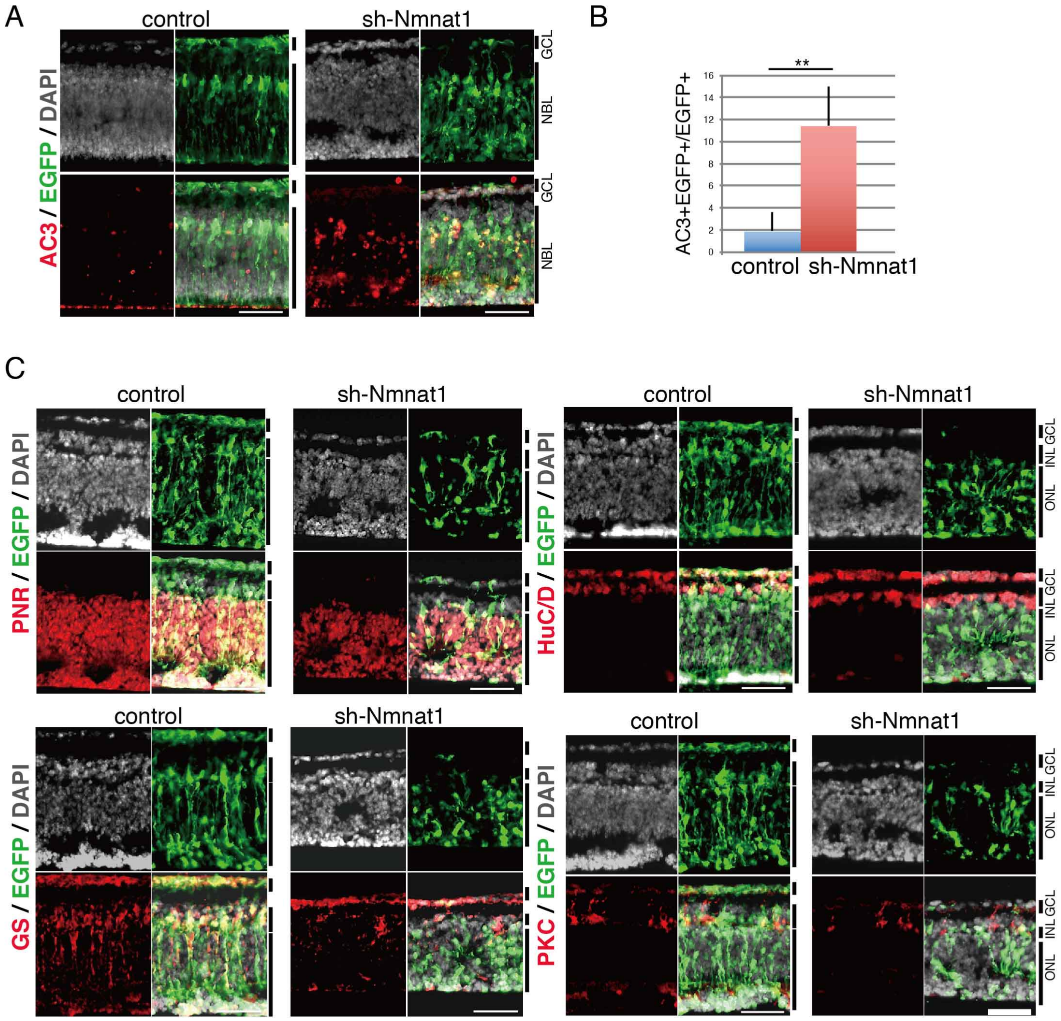

A-C. Isolated retinas at P1 were transfected with control or sh-Nmnat1 plasmid with EGFP-expressing plasmid, and after 4 days (A), or 10 days (C), retinas were harvested and immunostained with anti-EGFP and indicated antibodies. Nuclei were visualized by staining with DAPI. AC3+EGFP+ double positive cell population in total EGFP positive cells are calculated. Average value of three independent samples with standard deviation are shown in B. Statistical calculation was performed by Student’s T-Test, p, **<0.01.

Supplemental Figure 6

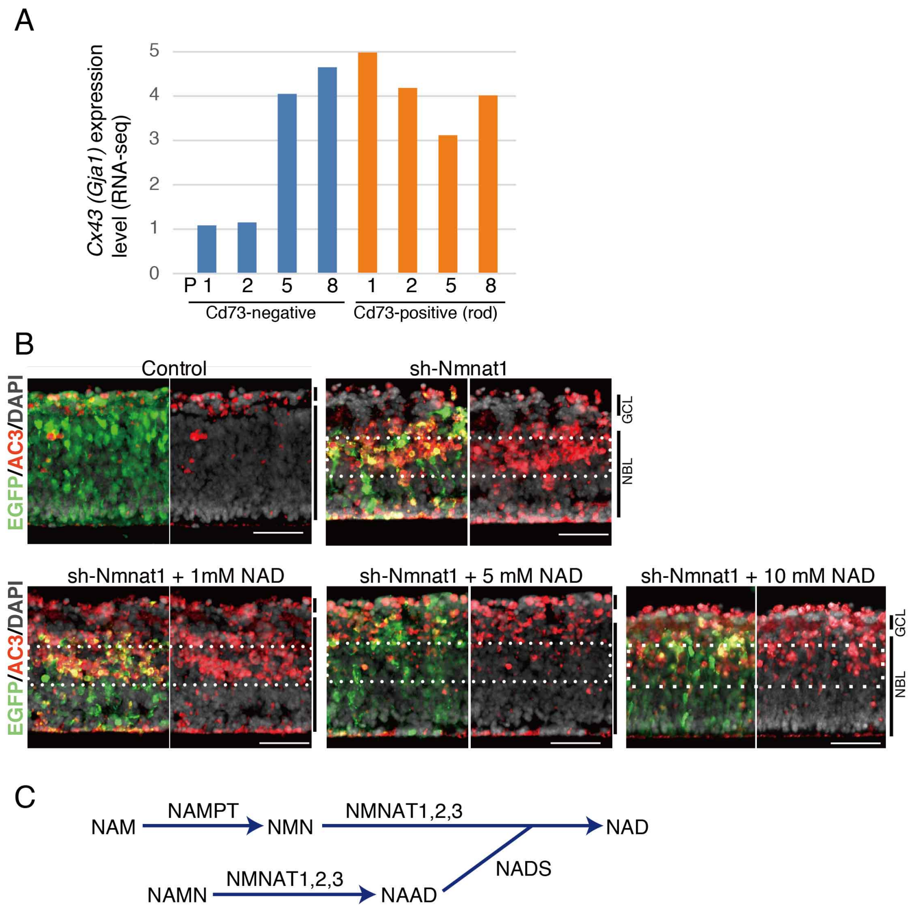

A. Expression levels of *Cx43 (Gja1)* transcript in developing retina (Cd73+ rod photoreceptors and Cd73- other cells) examined by RNA-seq are shown. B. Isolated retinas at E17.5 were transfected with control or sh-Nmnat1 and cultured as explants in the presence of NAD with indicated concentration. After 4 days, immunostaining was done by using anti-EGFP, -AC3 antibodies. Nuclei were visualized with DAPI. C. Schematic representation of Nmnats and NAD related derivatives.

Supplemental Figure 7

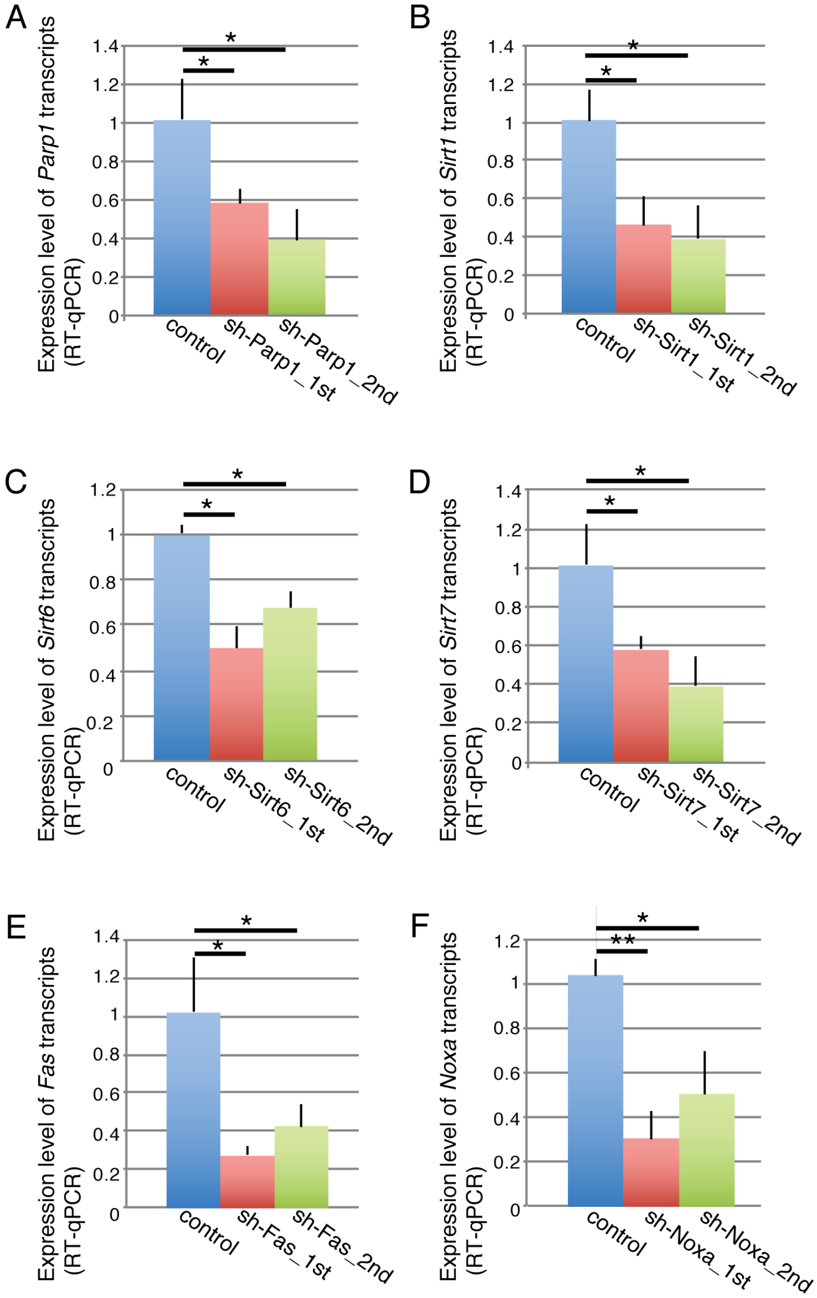

A-F. Plasmids encoding control, or indicated sh-RNA were transfected into NIH3T3 cells, and the cells were harvested after 48 hours of culture. RT-qPCR to detect indicated transcripts was done. Values are expressed relative to value of *Gapdh*. Three independent transfection were done, and averages with standard deviation are shown. Statistical calculation was performed by Turkey HSD, p, *<0.05, **<0.01.
