# Supplementary material for: Roles of Nmnat1 in the survival of retinal progenitors through the regulation of pro-apoptotic gene expression via histone acetylation
Source: Cell Death Dis. 2018 Aug 30;9(9):891. doi: 10.1038/s41419-018-0907-0 (PMC6117278; doi:10.1038/s41419-018-0907-0)
Supplement: Supplementary file 2 — Supplemental Tables [file 41419_2018_907_MOESM2_ESM.docx]

***Supplemental Table 1***

| transcript | forward primer | reverse primer |
| --- | --- | --- |
| Nmnat1 | 5’-gaaattgctgtgtggggcag-3’ | 5’-ccacgatttgcgtgatgtcc-3’ |
| Nmnat2 | 5’-gatgttcgagagagccaggg-3’ | 5’-aaggccctgttttccgtagg-3’ |
| Nmnat3 | 5’-aagacaccatcagcctctgc-3’ | 5’-ccaagccgaacttctccact-3’ |
| Sirt1 | 5’-agttccagccgtctctgtgt-3’ | 5’-gatcctttggattcctgcaa-3’ |
| Sirt6 | 5’-ggctacgtggatgaggtgat-3’ | 5’-ggctcagccttgagtgctac-3’ |
| Sirt7 | 5’-gactgagcgtactgcccttc-3’ | 5’-acaatggtatcccgaagctg-3’ |
| Parp1 | 5’-ggcaagcacagtgtcaaagg-3’ | 5’-tgtcgttgacaccagatggg-3’ |
| p53 | 5’-ccatggcccctgtcatcttt-3’ | 5’-tgaggggaggagagtacgtg-3’ |
| Bax | 5’-ctggatccaagaccagggtg-3’ | 5’-gtgaggactccagccacaaa-3’ |
| Bcl-xl | 5’-aggggcttagctgctgaaag-3’ | 5’-gtggacaaggatcttggggg-3’ |
| Bnip3 | 5’-aggcgtctgacaacttccac-3’ | 5’-cccaaggaccatgctagctc-3’ |
| Noxa | 5’-agtcgtggagctagggaagt-3’ | 5’-acgactgcccccatacaatg-3’ |
| Foxo1 | 5’-gcgtgccctacttcaaggat-3’ | 5’-gaagggacagattgtggcga-3’ |
| Apf1 | 5’-tgttttctcccgatggctcc-3’ | 5’-tgctttagcacgatggcaga-3’ |
| Fasl | 5’-aggaggtctgtgactgaggg-3’ | 5’-agtagacccaccctggaagt-3’ |
| Fas | 5’-tgctggctcacagttaagagtt-3’ | 5’-tcaggttggcatggttgaca-3’ |
| Tnfrsf1a | 5’-gctgttgcccctggttatct-3’ | 5’-atggagtagacttcgggcct-3’ |
| Tlr4 | 5’-agggtcacagaattctcagcg-3’ | 5’-tgttgagactggtcaagcca-3’ |
| Fadd | 5’-tgctccacctatccaccaga-3’ | 5’-caatgcggaaggcgattgag-3’ |
| Gapdh | 5’-tgaccacagtccatgccatc-3’ | 5’-cataccaggaaatgagcttgac-3’ |

***Supplemental Table 2***

| Target sequences for shRNA mediated gene knockdown | |
| --- | --- |
| shRNA | Target sequences |
| Scramble | 5’-aaggtatcgcaattaatggacgc-3’ |
| Nmnat1_1st | 5’-aaggactatatgcatgctacagg-3’ |
| Nmnat1_2nd | 5’-aagatggaggacatcacgcaaat-3’ |
| Sirt1_1st | 5’-aagaaaagatatcaatacaattg-3’ |
| Sirt1_2nd | 5’-aagcaacaaacaacaatgttaat-3’ |
| Sirt6_1st | 5’-aagctcccaatgcaataaataca-3’ |
| Sirt6_2nd | 5’-gacgatgtcggtgaattat-3’ |
| Sirt7_1st | 5’-aagtgtgatgatgtcatgcaact-3’ |
| Sirt7_2nd | 5’-aagtcttattaaacatctctcaa-3’ |
| Parp1_1st | 5’-aagttaatttcatactatataga-3’ |
| Parp1_2nd | 5’-aagaaatgcagcgagagtattcc-3’ |
| Fas_1st | 5’-aagaaccattatgctgataaatg-3’ |
| Fas_2nd | 5’-aagagactataactcatctctat-3’ |
| Noxa_1st | 5’-aagtaactagtaataatctatct-3’ |
| Noxa_2nd | 5’-aagaaatgaaccaccttaaatcc-3’ |

***Supplemental Table 3***

| Primer sequences used for ChIP-qPCR | | |
| --- | --- | --- |
| Target gene locus | Forward Primer | Reverse Primer |
| Fas #1 | ATAGGAGCGAAGCGGTTTGT | CAGCTCTAGACTGCCTGTGG |
| Fas #2 | TTGGGGCTTTACGAAGATTG | TCAGGTCTTCCCAGAAATCG |
| Fas #3 | TATGCAATCAAGCCCTGCTT | CAAGGGTTGCGAGAAAGTTC |
| Fas #4 | AGCAATCAGCCAAAGTCCAA | ATTGCATAAATGGGCATTCC |
| Fas #5 | TCCTAGGGTGTGTACCTCCT | GGAGACAAAGACCATTTTGC |
| Fas #6 | TTCATTCTCCATTTGGGAAA | GATTCTGATGAGGGAGGTCC |
| Fas #7 | CCCCCGTGGTTCTAAAGATT | TCCCAAATGGAGAATGAAGG |
| Fas #8 | TTCATCGTCTTCCTTTCCCC | AATCTTTAGAACCACGGGGG |
| Noxa #1 | GGATGTCGTCACATGACGTC | ATCCTCTCTGTTCAGGCGCC |
| Noxa #2 | CTCGAGACCTGCTCCACTTC | GACGTCATGTGACGACATCC |
| Noxa #3 | GTTGAGCAGGACTCGTCCTC | AAGTGGAGCAGGTCTCGAGA |
| Noxa #4 | CCCAGCAATGGATACGATCT | GAGGACGAGTCCTGCTCAAC |
| Noxa #5 | GTCCCTAATTGGCGAAGAAT | AGATCGTATCCATTGCTGGG |
| Noxa #6 | TGCTCGAACTTGGGACTCTT | ATTAGGGACGAACCCCAGAA |
| Noxa #7 | CTTCTGCCTAGGGCTGACAC | AAGAGTCCCAAGTTCGAGCA |

***Supplemental Table 4***

| Plasmid amount for loss of function analysis of Nmnat1 | | | |
| --- | --- | --- | --- |
|  | pU6-Scramble | pU6-shNmnat1 | pCAG-EGFP |
| Control | 80μg | - | 20μg |
| shNmnat1 | - | 80μg | 20μg |

| Plasmid amount for loss of function analysis of Sirt1, -6, -7 and Parp1 | | | | | | |
| --- | --- | --- | --- | --- | --- | --- |
|  | pU6-  Scramble | pU6-  shSirt1 | pU6-  shSirt6 | pU6-  shSirt7 | pU6-  shParp1 | pCAG-  EGFP |
| Control | 80μg | - | - | - |  | 20μg |
| shSirt1 | - | 80μg | - | - |  | 20μg |
| shSirt6 | - | - | 80μg | - |  | 20μg |
| shSirt7 | - | - | - | 80μg |  | 20μg |
| shParp1 |  |  |  |  | 80μg | 20μg |

| Plasmid amount for rescue experiment by co-electroporation of shFas or shNoxa with shNmnat1 | | | | | |
| --- | --- | --- | --- | --- | --- |
|  | pU6-  Scramble | pU6-  shNmnat1 | pU6-  shFas | pU6-  shNoxa | pCAG-  EGFP |
| Control | 80μg | - | - | - | 20μg |
| shNmnat1 | 30μg | 50μg | - | - | 20μg |
| +shFas | - | 50μg | 30μg | - | 20μg |
| +shNoxa | - | 50μg | - | 30μg | 20μg |

| Plasmid amount for in vivo electroporation | | | |
| --- | --- | --- | --- |
|  | pU6-Scramble | pU6-shNmnat1 | pCAG-EGFP |
| Control | 4μg | - | 1μg |
| shNmnat1 | - | 4μg | 1μg |
